# Sex-Specific Association between Fasting Plasma Glucose and Serum Selenium Levels in Adults from Southern Mexico

**DOI:** 10.3390/healthcare10091665

**Published:** 2022-08-31

**Authors:** María Judith Rios-Lugo, Ana Gabriela Palos-Lucio, Claudia Inés Victoria-Campos, Angel Lugo-Trampe, Karina Del Carmen Trujillo-Murillo, Maximiliano Arahon López-García, Marisol Espinoza-Ruiz, Elizabeth Teresita Romero-Guzmán, Héctor Hernández-Mendoza, Consuelo Chang-Rueda

**Affiliations:** 1 Sección de Medicina Molecular y Traslacional, Centro de Investigación en Ciencias de la Salud y Biomedicina (CICSaB), Universidad Autónoma de San Luis Potosí, Avda Sierra Leona 550, San Luis 78210, San Luis Potosí, Mexico; 2 Unidad de Posgrado, Facultad de Enfermería y Nutrición, Universidad Autónoma de San Luis Potosí, Avda. Niño Artillero 130, San Luis Potosí 78210, San Luis Potosí, Mexico; 3 Facultad de Medicina Humana, Campus IV, Universidad Autónoma de Chiapas, Carretera a Puerto Madero Km 1.5, Tapachula 30580, Chiapas, Mexico; 4 Facultad de Ciencias Químicas, Campus IV, Universidad Autónoma de Chiapas, Carretera a Puerto Madero Km 1.5, Tapachula 30580, Chiapas, Mexico; 5 Departamento de Química, Gerencia de Ciencias Básicas, Dirección de Investigación Científica, Instituto Nacional de Investigaciones Nucleares, Carretera Mexico-Toluca s/n, La Marquesa, Ocoyoacác 52750, State of Mexico, Mexico; 6 Instituto de Investigación de Zonas Desérticas, Universidad Autónoma de San Luis Potosí, Altair 200, San Luis 78377, San Luis Potosí, Mexico; 7 Universidad del Centro de Mexico, Capitán Caldera 75, San Luis 78250, San Luis Potosí, Mexico; 8 Hospital General de Soledad de Graciano Sánchez, Secretaría de Salud, Valentín Amador 1112, Soledad de Graciano Sánchez 78435, San Luis Potosí, Mexico

**Keywords:** serum selenium, cardiometabolic traits, fasting plasma glucose, Mexican adults

## Abstract

Selenium (Se) is an essential trace element that by its antioxidant properties has been studied to elucidate its participation in the development of obesity and type 2 diabetes. We evaluated the association between cardiometabolic traits and serum Se levels in a sample of adults from southern Mexico. In 96 nondiabetic individuals, anthropometric data and clinical biochemistry measurements were analyzed. Serum total Se levels were measured with inductively coupled plasma mass spectrometry (ICP-MS). Serum Se level in the whole sample was 10.309 ± 3.031 μg mL^−1^ and no difference between the women and men was observed (*p* = 0.09). Additionally, fasting plasma glucose (FPG) was significantly associated with serum Se level (β = −0.07 ± 0.03, *p* = 0.02, analysis adjusted for age, sex and BMI). Furthermore, sex shows significant interaction with FPG on the serum Se levels (*p* = 0.01). A follow-up analysis revealed the particular association between FPG and Se levels in women (β = −0.10 ± 0.04, *p* = 0.01). In conclusion, our data evidenced a women-specific association between FPG and serum Se levels in a sample of adults from southern Mexico.

## 1. Introduction

At least 90% of diabetes cases in the world are represented by type 2 diabetes (T2D) and its high relationship with overweight and obesity, is responsible to take an important place in the most critical public health concerns worldwide [1]. In Mexico, the last National Survey of Health and Nutrition (ENSANUT, 2020) reported the prevalence of obesity and total diagnoses of diabetes as 36% and 15.7%, respectively, in adults aged 20 years and over [2].

Diabetes is highly associated with the risk to develop diseases related to the kidney, retina, nervous system, cardiovascular diseases (disorders of the heart and/or blood vessels) and premature death [3]. T2D is characterized by hyperglycemia as a consequence of systemic inflammation, oxidative stress decreased insulin sensitivity in adipose tissue, liver and muscle implicated in obesity, and reduced secretion of insulin as a result of pancreatic β cell dysfunction [4]. Furthermore, it is known that the main risk factors for T2D are obesity, low physical activity and a high-calorie diet next to biological factors, such as sex, age, gut microbiome, epigenetic and genetic contributions [3,5]. However, selenium (Se) is an essential trace element that has been studied to elucidate its participation in the development of obesity and T2D [6,7,8,9,10,11]. Se is a key component of glutathione peroxidases (GPx), which are selenoproteins that control the cell redox status to protect it from oxidative stress. In vivo and in vitro studies suggest that Se could mediate insulin sensitivity. Se is also a key component of selenoproteins P and thioredoxin reductases, which have been found in association with impaired glucose metabolism markers [12,13,14].

Studies performed in adult populations from the United States, Asia and Europe have reported significant associations between serum Se concentration, body mass index (BMI), waist circumference, body fat percentage and obesity risk [15,16,17,18]. However, this association has not reached statistical significance in all the Asian and European studies [19,20,21]. Additionally, in vivo and in vitro studies have reported an antidiabetic effect of Se. Nevertheless, results from epidemiologic and follow-up studies are not conclusive. In adults from Croatia, Spain and France the high concentration of Se has been associated with low frequency of diabetes and decreased risk of hyperglycemia [22,23,24]. In contrast, in the United States population, the serum Se levels have shown a positive association with diabetes risk or no effect on T2D risk after supplementation [25,26]. Concerning the association between serum Se levels and fasting glucose, recent studies from Asia and Europe report that increased serum Se levels are positively associated with elevated fasting glucose levels [27,28,29,30].

The association between serum Se and glucose levels is controversial. However, environmental and biological factors could influence Se status and its association with glucose levels. For example, geographic differences regarding soil conditions and agricultural practices are conditioning factors of Se consumption and its status in different populations around the world [31]. On the other hand, due to the possible role of sex hormones in diabetes and the pathophysiology of metabolic diseases, recent studies have suggested exploring sex-specific associations of trace elements and metal exposure with diabetes risk and FPG level [29,32].

The relationship of serum Se concentration with obesity and T2D risk has been little explored in the Mexican population. In addition, due to the high prevalence of overweight, obesity and diabetes in Mexico, it is necessary to contribute scientific evidence to improve strategies to treat and prevent these metabolic diseases. For that, the aim of the present study was to evaluate the association of cardiometabolic traits with serum Se levels in a sample of adults from southern Mexico.

## 2. Materials and Methods

### 2.1. Study Sample and Ethical Approval

The study included 96 Mexican adults (42 women and 54 men) without T2D from Tapachula, Chiapas, Mexico. Serum samples and anthropometric data collected were performed from September 2016 to February 2018. The protocol was carried out according to the relevant guidelines and regulations established in the Declaration of Helsinki and was approved by the Institutional Review Board of the Tapachula School of Human Medicine of UNACH (03/MHT/RPR/087/17). Prior to registering for the study, all the participants signed approved consent forms. By self-report, adults with diseases that could confuse the association analysis in the study, such as AIDS and/or chronic liver/kidney disease and/or any cancer, were excluded from the study.

### 2.2. Anthropometric and Clinical Measurements

The measurements of weight and height were conducted by professionally trained staff with a clinical stadiometer (Clínica-160, Básculas Nuevo León^®^, Monterrey, NL, Mexico) and the body mass index (BMI) was calculated as weight (kg)/height (m^2^). According to BMI criteria of the World Health Organization (WHO), normal weight, overweight and obesity status were determined as having a BMI of 18.5–24.9 kg/m^2^, BMI 25–29.9 kg/m^2^ and BMI ≥30 kg/m^2^, respectively. Systolic and diastolic blood pressure (SBP and DBP) were measured using a mercurial sphygmomanometer (ALPK2, Tokyo, Japan).

After at least 8 h of fasting, blood samples were obtained from all the participants and the serum was extracted to analyze the cardiometabolic traits. Fasting plasma glucose (FPG), total cholesterol (TC), high- and low-density lipoprotein cholesterol (HDL-C and LDL-C) and triglycerides (TG) were measured in a ChemWell^®^ 2910 Automated EIA and Chemistry Analyzer (Awareness Technology Inc., USA) by enzymatic colorimetric assay, while fasting plasma insulin (FPI) was determinate by enzyme-linked immunosorbent assay (Calbiotech Inc., USA). The homeostatic model assessment of insulin resistance (HOMA-IR) was calculated applying the Matthews et al. equation [33].

### 2.3. Serum Se Level Quantification by ICP-MS

The Se level quantification was performed with inductively coupled plasma mass spectrometry (ICP-MS iCAP Q, Thermo Scientific, Germany) and collision mode using He cell gas and kinetic energy discrimination (KED); 0.1 mL of serum was used in each sample treatment, which was traced with indium (In), following the protocol proposed by Rios-Lugo et al. [34]. Samples were digested by a microwave system (MARS6 CEM, Matthews, NC, USA) with 8 mL nitric acid for 15 min at a constant temperature (210 °C) following recovery and evaporation to dryness of the samples. Finally, samples were diluted to 10 mL with 2% *v*/*v* nitric acid for Se analysis by ICP-MS with an external calibration curve of Se (0.01, 0.05, 0.1, 0.5, 1, 5, 10, 25 and 50 μg L^−1^). For all calculations, this study was considering the final volume (10 mL), serum volume (0.1 mL), blanks of samples and recovery of an internal standard of In (1 ng mL^−1^). Concentrated high-purity nitric acid (Milestone Duopur system Milestonesrl, Italy) and high-purity water with >18 MΩ cm (Milli-Q^®^ system Millipore, Mexico) were used in all processes. The Se standard used in this study was obtained from the High-Purity Standards.

### 2.4. Statistical Analysis

The comparison of continuous variables and frequencies between women and men groups was performed with Student’s t and Chi-square, respectively. Using the row data and the one-way ANOVA test, we compared the serum Se levels between the normal weight, overweight and obesity groups. We evaluated the normality distribution of quantitative variables with the Shapiro–Wilk test and the rank-based inverse normal transformation [35] was employed to normalize the variables that did not reach a normal distribution (Appendix A). With the row data, we evaluated trough Spearman correlation for the simple association between the cardiometabolic traits and serum Se levels. The significant correlations were evaluated with linear regression models adjusted for age and sex, and additionally for body mass index. The interaction effect between the cardiometabolic traits, sex and body weight categories on the serum levels of Se was evaluated with a linear regression adding an interaction term and adjusting for age and sex, and additionally for body mass index [36]. The SPSS (version 22.0, IBM, Armonk, NY, USA) was used to perform the statistical analysis. A two-sided p-value <0.05 was considered significant in the association analysis.

## 3. Results

### 3.1. Description of the General Characteristics of the Study Sample

The general characteristics of the study sample are presented in Table 1. The mean serum Se level in the whole sample was 10.309 ± 3.031 μg mL^−1^ and no difference was observed between the women and men groups (Mean difference = 1.03 µg dL^−1^, *p* = 0.09; Table 1). All the anthropometric and clinical variables were similar between women and men groups (*p* ≥ 0.09), except HDL-C level, which was significantly higher in women than in men (*p* = 1.0 × 10^−3^), while DBP and SBP, and the level of TG was significantly lower in women (*p* ≤ 0.01).

### 3.2. Association between BMI, Overweight, Obesity and Serum Se Level

**Figure 1:**
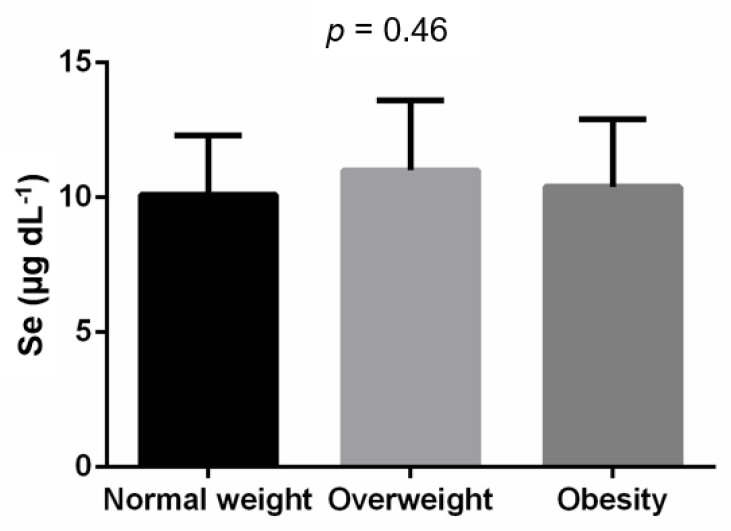
Comparison analysis of serum Se levels between body weight categories. Sample size: Normal weight = 34; Overweight = 19; Obesity = 43. Analysis by one way ANOVA.

We did not find significant association between BMI and serum Se level in the correlation analysis (*r* = 0.09; *p* = 0.37; Table 2). No differences were observed when comparing the serum Se levels between individuals with normal weight, overweight and obesity (*p* = 0.464, Figure 1).

### 3.3. Association between Serum Se Level and Cardiometabolic Traits

**Figure 2:**
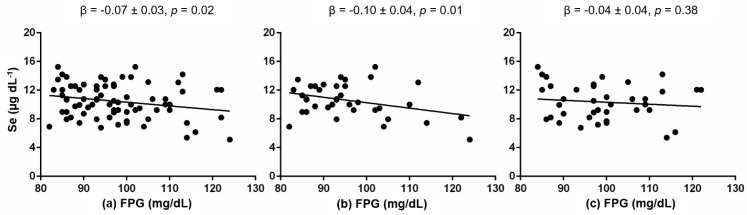
Association between fasting plasma glucose (FPG) and serum Se levels in the whole sample (**a**) and separately in women (**b**) and men (**c**). Analysis by linear regression model adjusted for age, sex and body mass index (whole sample), and for age and body mass index (separately in women and men).

First, we performed a correlation analysis between the cardiometabolic traits and serum Se level (Table 2). Only FPG was significantly correlated with serum Se level (*r* = −0.24, *p* = 0.02). FPG continued significantly associated with serum Se level in a linear regression model adjusted for age, sex and BMI (*β* = −0.07 ± 0.03, *p* = 0.02; Figure 2).

With an interaction analysis, we evaluated if sex or body weight could modify this association. Sex shows a significant interaction between FPG and serum Se levels (*p* = 0.01). The interaction analysis between body weight categories and FPG on the serum Se levels was not significant (*p* = 0.76). We then tested the association between FPG and serum Se levels separately in women and men groups (Figure 2). We only observed a significant negative association between FPG and serum Se levels in women (*β* = −0.10 ± 0.04, *p* = 0.01; Figure 2). This association was not significant in the men group (*p* = 0.38; Figure 2).

## 4. Discussion

The present study evaluated the association of cardiometabolic traits with serum Se levels in a sample of Mexican adults without diabetes. Our results show a women-specific association between FPG and serum Se levels.

While a significant association between serum Se levels and markers of overweight and obesity has been reported in Americans, Asians and Europeans [15,16,17,18,37,38], our results did not replicate this association. With the exception of FPG, no association between the rest of the cardiometabolic traits, obesity and serum Se levels, was observed. Among the possible risk factors that could be influencing the association between cardiovascular traits and serum Se concentration, we can mention tobacco and alcohol intake, which could be related to the absorption and metabolism of Se and may affect its basal concentration in serum [19]. On the other hand, due to the Se content of the soil, pH and organic-matter content, there are wide geographic differences in Se intake and in consequence, differences in body Se status [39]. As an illustration, countries in Asia, Europe, North America, and South America have reported an average Se intake of 2.6–48 µg/day, 10–60 µg/day, 80–221 µg/day, and 80–500 µg/day, respectively [31]. Although in Mexico it is still necessary to publish reliable information related to the Se content of the soil, it has been reported that there is an average Se intake of 37.6 ± 51.8 µg/day in the adult population, without differences between sexes [40]. Additionally, the adult population from northern Mexico has found that beans, corn tortillas and milk are responsible for 32%, 24%, and 19% of the daily Se intake, respectively [40,41]. On the other hand, although ICP-MS is a reliable method widely used to quantify Se [42,43], and we carry out the Se measurement process with strict quality control, to date, there is no consensus regarding which biological sample is the most suitable for measuring Se status. It has only been reported that measurements on whole blood, serum, or toenails served reasonably well as a measure for ranking subjects according to long-term selenium intake [44,45], which leaves us with a wide field of research to continue exploring the trace elements status in the Mexican population.

Although the antidiabetic role of Se remains controversial, our results are in line with previous studies conducted on adults from Europe, the United States and Mexico [22,23,24,46,47]. These reports evidence that the serum Se concentrations in diabetic patients (noninsulin-dependent and insulin-dependent) are lower in comparison with healthy adults from Spain, Croatia and Mexico [23,24,47]. Similarly, a strong negative association between serum Se levels and diabetes risk was reported by Park et al. in the adult population of the United States [47]. Additionally, Akbaraly et al. in a 9-year follow-up study evidenced a protective effect of higher Se status at baseline on the later occurrence of hyperglycemia [24]. A possible biological mechanism to explain the negative association between serum Se levels and diabetes is the participation of Se in the functionality of GPx [48]. GPx are antioxidant enzymes that promote the redox balance eliminating molecules of hydrogen peroxide (H_2_O_2_) from the cell. In this way, Se could protect against insulin resistance through the GPx activity and the decreasing H_2_O_2_ concentration. Moreover, the reduction of H_2_O_2_ concentration contributes to a reduced activity of protein tyrosine phosphatase 1B, which is highly associated with dephosphorylation of the b-subunit of the insulin receptor and insulin receptor substrate 1 [49,50].

Until now, the biological mechanism to explain the relationship between Se and increases in diabetes risk is highly speculative. Animal model studies have reported that Se could disrupt insulin signaling, via selenoproteins [51,52]. As an illustration, increased selenoproteins P have been found in association with impaired insulin secretion and signaling. The administration of selenoproteins P in normal mice, through the inhibition of AMPK activity, result in hyperglycemia during a glucose tolerant test [12]. In mouse models, it has been reported that the suppression with polyclonal antibodies against selenoproteins P improves glucose intolerance and insulin secretion [13]. Furthermore, it has been proposed that reactive oxygen species (specifically H_2_O_2_) participate in the control of insulin signaling [53,54]. In cell culture models, H_2_O_2_ activates tyrosine phosphorylation cascades, an important cellular mechanism related to insulin signaling [53]. In this way, the overexpression of GPx1 as a consequence of the high concentration of Se could affect the insulin function through the over-quenching of intracellular reactive oxygen species required for insulin sensitizing [52,55].

The evidence for a sex-specific association between Se and metabolic diseases has been increasing in the literature. While some studies report significant Se concentration between women and men [19], other studies report a sex-specific association of Se with cancer risk, cognitive performance, serum lipid levels and first total and ischemic stroke risk [56,57,58,59]. Regarding the specific association of Se with T2D and insulin resistance, a piece of recent evidence shows that Se is independently associated with T2D in adult women with a mean level of Se of 136.4 ± 19.6 μg/L in the United States [60]. Later evidence in the French population also shows a sex-specific association between higher plasma Se concentration and lower risk of developing dysglycemia after nine years following in men with baseline plasma Se mean levels of 1.08 ± 0.21 μmol/L [24]. Furthermore, in Taiwanese adults with a mean serum Se concentration of 96.34 ± 25.90 μg/L, it was positively correlated with HOMA-IR only in women [29].

A biological mechanism to explain the women-specific association between FPG and serum Se levels found in this study is not clear yet. However, differences in the protein concentration and gene expression of GPx and selenoproteins P have been reported between men and women in animal models and humans [12,61]. On the other hand, differences in metabolic risk factors in men and women have been previously reported [62]. There are significant sex differences for metabolic diseases, from delayed disease onset to a higher prevalence of comorbid diseases for females [63]. In addition, sex differences regarding sensitive and biological responses to societal stress have been reported in association with the pathophysiology of IR and T2D [64,65,66]. On the other hand, prenatal and postnatal female hormones may play an important role in the Se metabolism. In this context, future studies would be interesting to evaluate if sex hormones mediate the association between Se and cardiometabolic traits. Additionally, the recent report about the sex-specific association between serum zinc and LDL-C levels in a Mexican population [67], encourage sex-specific analysis for further observational, cohort, and clinical trial studies, to offer better insight into the role of trace elements in the physiopathology and the potential proposal of personalized biomarkers for the early prevention and treatment of metabolic diseases.

Although our study is one of the first pieces of evidence for a women-specific association between FPG and Se in the Mexican population, we acknowledge that it presents some limitations. We recognize that the inclusion of demographic data and personal habits, such as tobacco smoking and alcohol consumption could increase the relevance of our results. Another limitation is that our study sample included individuals without diabetes, and due to the reduced sample size, we did not categorize them into normoglycemic and prediabetes groups. Nevertheless, this points out the importance of carrying out a comprehensive study to evaluate the state of Se in the Mexican population with prediabetes, type 2 diabetes and healthy controls, since, as was mentioned above, these conditions the state of oxidative stress and the metabolic state in general, which at the same time could relate to with Se levels. In addition, we only analyzed a population from the South of Mexico. Therefore, our results about the sex-specific association between serum FPG and serum Se levels need to be extended to children and adult populations and confirmed in large and more diverse Mexican cohorts. Our study also highlights the importance of carrying out multidisciplinary research to evaluate the content in the soil, the intake and the body status of Se and its relationship with biological (age and genetic and epigenetic aspects) and environmental factors that could mediate its association with the development of metabolic diseases in the Mexican population.

## 5. Conclusions

Our results evidence a negative association between serum Se levels and FPG in women from southern Mexico. However, additional studies need to be performed in large Mexican cohorts to confirm our findings.

## Figures and Tables

**Table 1:** General characteristics of the total sample.

Variable	Women (*n* = 42)	Men (*n* = 54)	*p*-Value
Age (years)	46.69 ± 11.62	50.51 ± 10.39	0.09
BMI (kg/m^2^)	28.35 ± 8.22	30.19 ± 6.51	0.22
SBP (mmHg)	109.47 ± 14.98	123.09 ± 13.97	**1.0 × 10^−5^**
DBP (mmHg)	71.79 ± 9.17	78.52 ± 9.23	**4.7 × 10^−4^**
TC (mg/dL)	191.70 ± 36.73	180.81 ± 36.65	0.18
HDL-C (mg/dL)	49.24 ± 12.39	39.81 ± 11.43	**1.0 × 10^−3^**
LDL-C (mg/dL)	116.09 ± 31.72	106.56 ± 32.46	0.19
TG (mg/dL)	129.70 ± 61.20	177.95 ± 101.28	**0.01**
FPG (mg/dL)	95.56 ± 10.26	99.31 ± 10.21	0.10
FPI (µU/mL)	8.48 ± 7.15	9.51 ± 6.16	0.45
HOMA-IR	2.01 ± 1.80	2.68 ± 2.70	0.21
Se (µg dL^−1^)	10.87 ± 3.11	9.84 ± 2.90	0.09
Overweight *n* (%)	8 (19.0)	11 (20.4)	0.32
Obesity *n* (%)	16 (38.1)	27 (50.0)	

Data are expressed as mean ± standard deviation or n (%). Student t-tests and Chi-square were used to compare means and frequencies. Abbreviations: BMI, body mass index; SBP, systolic blood pressure; DBP, diastolic blood pressure; TC, total cholesterol; HDL-C, high-density lipoprotein cholesterol; LDL-C, low-density lipoprotein cholesterol; TG, triglycerides; FPG, fasting plasma glucose; FPI, fasting plasma insulin; HOMA-IR, homeostatic model assessment for insulin resistance; Se, selenium. Significant *p*-values (*p* < 0.05) are represented in bold.

**Table 2:** Correlation between serum Se level and cardiometabolic traits.

Variable	Spearman Correlation Coefficients
BMI (kg/m^2^)	0.09 (0.37)
SBP (mmHg)	−0.13 (0.18)
DBP (mmHg)	−0.14 (0.15)
TC (mg/dL)	0.06 (0.59)
HDL-C (mg/dL)	0.11 (0.30)
LDL-C (mg/dL)	0.149 (0.18)
TG (mg/dL)	−0.02 (0.84)
FPG (mg/dL)	**−0.24 (0.02)**
FPI (µU/mL)	−0.15 (0.14)
HOMA-IR	−0.16 (0.15)

Data are expressed as Pearson’s coefficient (*p*-value). Abbreviations: BMI, body mass index; SBP, systolic blood pressure; DBP, diastolic blood pressure; TC, total cholesterol; HDL-C, high-density lipoprotein cholesterol; LDL-C, low-density lipoprotein cholesterol; TG, triglycerides; FPG, fasting plasma glucose; FPI, fasting plasma insulin; HOMA-IR, homeostatic model assessment for insulin resistance; Se, selenium. Analysis by Spearman correlation. Significant p-values (*p* < 0.05) are represented in bold.

## Data Availability

Not applicable.

## Appendix A

**Table A1:** Normality evaluation.

Variable	Untransformed	Transformed
BMI (kg/m^2^)	**1.6 × 10^−8^**	0.999
SBP (mmHg)	0.044	0.999
DBP (mmHg)	0.101	-
TC (mg/dL)	0.263	-
HDL-C (mg/dL)	0.051	0.995
LDL-C (mg/dL)	0.384	-
TG (mg/dL)	**1.1 × 10^−13^**	0.999
FPG (mg/dL)	**0.004**	0.995
FPI (µU/mL)	**1.7 × 10^−8^**	0.998
HOMA-IR	**7.1 × 10^−9^**	0.999

Data are represented as Shapiro–Wilk Test p-value. Shapiro–Wilk tests were performed on BMI and metabolic outcomes before and after rank transformation to determine whether they are normally distributed. Abbreviations: BMI, body mass index; SBP, systolic blood pressure; DBP, diastolic blood pressure; TC, total cholesterol; HDL-C, high-density lipoprotein cholesterol; LDL-C, low-density lipoprotein cholesterol; TG, triglycerides; FPG, fasting plasma glucose; FPI, fasting plasma insulin; HOMA-IR, homeostatic model assessment for insulin resistance; Significant *p*-values (*p* < 0.05) are represented in bold.
